# Exploring the relationship between macroeconomic indicators and sectoral indices of Indian stock market

**DOI:** 10.12688/f1000research.160668.1

**Published:** 2025-02-10

**Authors:** Sanjay Singh Chauhan, Pradeep Suri, Bhekisipho Twala, Neeraj Priyadarshi, Farman Ali

**Affiliations:** 1 Uttaranchal Institute of Management, Uttaranchal University, Dehradun, Uttarakhand, 248007, India; 2 Digital Transformation Portfolio, Tshwane University of Technology, Staatsartillerie Road, Pretoria West, Pretoria, 0183, South Africa; 3 Department of Electrical Engineering, JIS College of Engineering, Kolkata, West Bengal, 741235, India

**Keywords:** sectoral Indices, Economic policy uncertainty, ARDL, National stock exchange

## Abstract

**Background of the study:**

The influence of macroeconomic indicators makes it important to study the relationship between macroeconomic indicators and stock market return. On further analysis it can be observed that different sectors respond differently to change in the macroeconomic indicator that is important for investors, researchers and policy makers.

**Methods:**

The autoregressive distributed lag (ARDL) model is applied to study influence of macroeconomic indicators on sectoral return of NSE from April 2012 to August 2024.

**Results:**

Findings of the study show that macroeconomic indicators influence sectoral return in the short run as well as long run and the influence is differential. The analysis of long run relationship shows that Foreign Institutional Investment (FII) significantly affects all the sectoral indices except IT. Index of industrial production (IIP) have significant relationship with Auto, IT, Media, Metal and Pharma. Money supply (MS) significantly affects Bank, FMCG and IT in the long run. Wholesale Price Index (WPI) has significant relationship with Auto, FMCG and Media in the long run. Economic Policy Uncertainty Index (EPU) affects Auto, FMCG and Pharma in the long run. Crude oil price (COP) has significant effect only on Media in the long run. Exchange rate (ER) does not have significant effect on any of the sectoral index.

**Conclusion:**

In the long run FII, IIP, EPU, MS and WIP are major determinants of stock market return. In the short run FII, ER and COP are major determinants of stock market return.

## Introduction

The performance of stock markets is closely connected to the overall economic environment, with macroeconomic variables significantly influencing investor psychological state, market trends, and sector performance. In emerging nations such as India, characterized by vigorous economic development and evolving market dynamics, comprehending the correlation between macroeconomic indicators and sectoral indices is essential for policymakers, investors, and analysts.

This research article seeks to examine the influence of significant macroeconomic variables, including Crude Oil Price (COP), Economic Policy Uncertainty Index (EPU), Exchange Rate (ER), Foreign Institutional Investment (FII), Wholesale Price Index (WPI), Index of Industrial Production (IIP) and Money Supply (MS) on the performance of different sectoral indices in the Indian stock market. The research aims to identify empirical correlations that demonstrate how variations in economic fundamentals affect sector-specific returns. Empirical research demonstrates that factors such as inflation, GDP growth, interest rates, exchange rates, and global economic conditions profoundly influence sectoral performance. The banking sector is acutely responsive to alterations in monetary policy, but the IT sector may be more influenced by global economic trends. Research employing methodologies like as Vector Autoregression (VAR), Dynamic Conditional Correlation-GARCH (DCC-GARCH), and wavelet techniques has illuminated volatility patterns and resilience in Indian markets ( Narayana Maharana *et al.*, 2024). Moreover, research indicates that sectoral indexes on the National Stock Exchange (NSE) exhibit clustered volatility and leverage effects, implying nonlinear dependence on macroeconomic variables ( Yalgi & Sinha, 2024).

Mohnot *et al.* (2024) identified a substantial correlation between all macroeconomic factors and the stock market index of Malaysia. The cointegration findings demonstrate a long-term association, while the vector autoregressive impulse response analysis indicates that the Malaysian stock index (KLCI) reacts adversely to the money supply, inflation, and producer pricing index (PPI).

Several researchers have examined the correlation between exchange rates and stock prices ( Chkili & Nguyen, 2014; Dahir *et al.*, 2018; Huang *et al.*, 2021; Hussain & Bashir, 2013; Mohnot *et al.*, 2024). According to the literature, there are two categories of theories regarding these two variables. This initial explanation, referred to as the “Flow Oriented Model” or “Good Market Approach,” contrasts with the “Stock Oriented Model” or “Portfolio Balance Approach” that offers a second explanation. According to Dornbusch and Fischer (1980), stock prices are influenced by exchange rates. A significant factor that affects trade competitiveness is fluctuations in exchange rates. Actual output and stock prices are influenced by trade competitiveness ( Bashir *et al.*, 2016; Hussain & Bashir, 2013; Pan *et al.*, 2007; Rai & Garg, 2022).

An exchange rate is the value at which one country’s currency, such as the Indian rupee, is traded for the currencies of other nations, such as the US dollar and euro ( Nigussie, 2016). In the twentieth century, most governments worldwide utilized fixed exchange rates for their currencies rather than allowing market forces to dictate them. The currencies of the countries involved in World War I were established based on gold valuation. During World War II, the designated monetary system underwent another transformation. The value of most countries’ currencies is fixed in terms of the US dollar, with the equilibrium exchange rate established by the foreign exchange market through the equilibrium of currency demand and supply ( Aguirre & Calderón, 2005). Most countries’ currencies were linked to the British Sterling Pound through the Bretton Woods system until 1970; however, following the collapse of this system, the exchange rate regime shifted across numerous nations. Most countries implemented a floating exchange rate but restricted the nominal exchange rate’s unrestricted movement until they became apprehensive about floating ( Sallenave, 2010). The central bank of the globe was tasked with sustaining the exchange rate, as nations implemented a fixed exchange rate system. The exchange rate was established by the buying and selling of currencies to sustain demand and supply in currency markets. In 1973, the floating exchange rate was established as nominal, which investors found unappealing due to the risks associated with exchange rate volatility. Following World War II, American dominance expanded in the global marketplace. In 1971, the majority of nations pegged their currencies to the US dollar. The currency devaluation was influenced by political interactions and instability ( Parveen *et al.*, 2012).

Research on forecasting crude oil prices has persisted for decades, during which numerous approaches have been proposed. Alongside conventional econometric methods, diverse machine learning techniques are employed to extract the intrinsic complexity of oil prices. Neural networks and support vector machines (SVM) are among the most common machine-learning techniques employed to predict oil prices. Their ability to replicate complex characteristics like non-linearity and volatility is well esteemed. In recent years, there has been a rise in the application of semi-supervised learning (SSL) ( Sen *et al.*, 2023) and gene expression programming (GEP) for predicting oil prices ( Khan *et al.*, 2024). This article analyses the varied impacts of macroeconomic factors on different businesses by examining sectoral indices, highlighting the distinct responses of sectors such as banking, pharmaceuticals, IT, and consumer goods. The findings from this study can be a beneficial resource for investors seeking to enhance their portfolios and for policymakers endeavoring to promote economic stability and progress.

This research utilizes econometric modelling and historical data analysis to enhance the existing literature on the relationship between macroeconomics and financial markets in emerging economies. The results are anticipated to provide strategic insights into market behaviour, facilitating informed decision-making in a complex and swiftly evolving financial environment. M. K. Khan *et al.* (2023) analysed a dynamic simulated autoregressive distributed lag model, revealing that oil and gold prices positively influence stock returns in both the short and long term, whereas the exchange rate exerts a negative impact in both time frames. The Indian stock market functions as a crucial indicator of the nation’s economic vitality and investor confidence. Sectoral indices, which denote specific market segments, provide critical insights into the performance of particular industries and illustrate the fundamental economic dynamics. Comprehending the correlation between macroeconomic factors and sectoral indices is essential for policymakers, investors, and market participants.

## Literature review

Financial markets and real economic activity are regularly impacted by institutional investments ( Bond *et al., *2012; Naik *et al., *2021; Nofsinger, 2005; Woolridge & Snow, 1990). Securities include debt instruments, commercial securities, shares, bonds, government securities, treasury bills and other derivatives of securities. However the stock market represents a portion of the capital market. Shares of publicly listed corporations can be traded on a stock market. Businesses can raise money on the primary market by issuing shares to the general public through an initial public offering (IPO). The part of the capital market where issuers directly issue and sell equity-backed securities is known as the primary market. Investors purchase securities that have never been traded. Only derivatives and stock transactions are performed on the stock exchange. According to Yalçın (2010), an efficient market is characterized by rapid information dissemination which is reflected in stock prices.

Presently, the National Stock Exchange offers fifteen sectoral indices. The sectoral index is a statistic that is regularly assessed and shows the total price movement of a selection of stocks chosen based on certain standards and procedures. Measuring market sentiment, developing derivative products and passive investment products such as Index Funds and Index ETFs, benchmarking active portfolios, representing asset classes in asset allocation, measuring and modelling returns on investment (return), systematic risk, and risk-adjusted performance are among the objectives and advantages of the sectoral index Suriani *et al. *(2024).

There exists a close relationship between stock market and macroeconomic indicators. To what extent is the stock market impacted by the macroeconomic indicators?

In 2007 Olowe, R. A. (2007) employed Johansen’s vector correlation model to examine the dynamic linkage between macroeconomic variables (Industrial production, Inflation, interest rate, money supply, exchange rate and oil prices) and stock price in Nigerian Stock Exchange and found that stock price is positively affected by inflation, money supply, Oil price and interest rate while negatively by exchange rate and industrial production. Mustafa, K. and Nishat, M. (2008) analyzed Pakistan’s stock market movement and macroeconomic variables (gold price, money supply, interest rate and exchange rate using error correlation model and granger trivariate technique which indicate existence of unidirectional association between stock prices and exchange rates. Gold prices, money supply and interest rates did not show a granger cause, while money supply and interest rates had a greater impact on stock prices. Brenner *et al. *(2009) studied the response of US share, Treasury bills and corporate bonds market to macroeconomic news and releases using GARCH DCC model and found that bond prices and share price are affected by the macroeconomic information. Oseni, I. O. and Nwosa, P. I. (2011) examined the response of the Nigerian stock market to economic factors like interest rate, GDP, and inflation. They found that GDP and stock price movements both had impact the market, but no impact was found due to change in interest rate and inflation. Zubair, A. (2013) analyzed the link between key economic factors and Nigeria’s stock market using Johansen’s cointegration and Granger-causality which indicated no relationship between the exchange rate and the All Share Index, and no causality between money supply and the stock market index during the crisis period. Kganyago, T. and Gumbo, V. (2015) examined relationship between Zimbabwe’s stock market returns and interest rates. Result of Johansen cointegration tests indicated a negative relationship between interest rates and share market returns. Additional Granger causality test findings demonstrated the existence of short-run causality between money market interest and stock market returns. Dutta, A. (2018) found a long-term association between oil prices and US energy sector stock market using ARDL bound tests. The US energy sector stock markets and the implied volatilities of oil have short-term “lead-lag” correlations, according to the Granger causality test. Rathnayaka and Seneviratna (2018) examined the impact of macroeconomic factors on Colombo Stock Exchange and found that macroeconomic factors directly affected stock market fluctuations, additionally real GDP and broad money supply significantly affect Colombo Stock Exchange.

Muradoglu *et al.* (2000) studied the causal relationship between stock market returns of 19 emerging nations and macroeconomic variables like interest rates, foreign exchange rates, inflation, and industrial production using Granger causality test and found that stock market size, global interaction, and financial liberalization influenced relationship between stock market and macroeconomic variables. Chaudhuri, K. and Koo, K. (2001) examined the impact of domestic and external factors on four Asian stock markets (India, S. Korea, Malaysia and Thailand) volatility. They found that national and global variables, such as IIP, exchange rate, government expenditure, and MS2, significantly influence stock returns. The study also revealed that government policies, including monetary and fiscal policies, were equally influential in the stock market. Muhammad *et al. *(2002) studied the long-run and short-run associations of stock prices and exchange rates in four South Asian countries using cointegration, error correction modelling, and standard Granger causality tests. The results indicate no long-run or short-run associations between variables in Pakistan and Indian and no short-run association for Sri Lanka and Bangladesh while existence of long run association is identified in case of Sri Lanka and Bangladesh. Chancharoenchai *et al. *(2005) studied the relationship between macroeconomic variables and stock returns in six Asian countries and found that macroeconomic factors have significant predictive power for excess returns. The results also indicates inefficiency in the stock market of the studied countries. Garza-García, J. G. and Vera-Juárez, M. E. (2010) studied the impact of Chinese and American macroeconomic factors on stock market indices of Mexico, Chile and Brazil using Cointegration, Granger causality, and VECM models. Results showed that macroeconomic factors of China and the US co-integrated with Latin American stock market. Moreover, Chinese macroeconomic factors Granger cause stock market indices of Chile and Mexico, but US macroeconomic variables Granger cause stock market indices of Brazil and Mexico. Hosseini *et al*. (2011) used Multivariate Cointegration and Vector Error Correction Model technique to study the relationship between major macroeconomic factors (crude oil price (COP), M2, IIP and the inflation rate) and stock market indices (India and China) and Found that crude oil price have a favorable effect in China but a negative effect in India. The money supply has a negative effect on the Indian stock market but a positive effect on stock market of China. Only in China does industrial output have a negative impact. Furthermore, rising inflation has a favorable impact on both stock indexes. Nasser *et al*. (2018) examined the relationship between macro-economic variables and five ASEAN countries stock market (Thailand, Philippines, Indonesia, Singapore, and Malaysia), using a generalized least square regression method, and found significant relationships between macroeconomic variables (unemployment rate; exchange rate; inflation rate; interest rate; GDP) and stock market of selected countries.

Pethe and Karnik (2000) examined the relationship between stock prices and macroeconomic variables such as the narrow money supply, IIP, wide money supply, lending rate, and exchange rate using econometric techniques like as the unite root test, cointegration, and error-correction models, indicating the absence of a long-term relationship between stock price and macroeconomic variables. Ahmad *et al. *(2006) studied weak form efficiency market hypothesis in Indian stock market revealing no normality and negative relationships among Indian stock market. Pal, K. and Mittal, R. (2011) analysed the long-term association between Indian capital markets and key economic factors such as exchange rate, inflation rate, interest rate, and Gross domestic saving (GDS) using the cointegration test and error correction mechanism (ECM) which indicates that exchange rate, inflation rate, interest rate has a significant impact whereas GDS is insignificant. Venkatraja, B. (2014) examined the relationship between the stock market and key macroeconomic indicators and found that 82% of changes in the BSE sensex were due to these variables, exchange rate, FII, IIP, and WPI having the most significant impact. Muthukumar *et al. *(2024) examined the impact of macroeconomic variables (exchange rate, gold price, inflation, interest rate) on Nifty 50 and found a significant relationship between the selected variables and Nifty 50. Further the results of the Granger causality test indicate that there is bidirectional causality between the inflation rate and the Nifty 50, but not between the interest rate, currency rate, and gold price. Hedau, A. (2024) Using logistic regression, researcher looked at macroeconomic factors influencing the performance of the NIFTY 50 index. They discovered that the Dow Jones index and changes in exchange rates are the primary drivers of the NIFTY 50 index. Experts, however, believe that predicting the movement of the NIFTY 50 index also requires consideration of other elements, such as political stability, the state of the developed, and India’s bilateral connections with other nations.

In 2002 Ewing, B. T. (2002) examined the relationship between NASDAQ Financial 100 index and macroeconomic factors, risk, inflation, real output and monetary policy using generalized impulse response analysis and found that monetary policy and inflation have negative effect on NASDAQ Financial 100 index whereas real output have positive effect. Tiwari, A. K. and Islam, F. (2012) examined the impact of portfolio diversification on investors’ gains in the stock market using data from eight BSE sectoral indices and found no common trend amoung sectoral indices in the long run indicating benefit of diversification within these indices. Pal, D. and Mitra, S. K. (2019) discovered a significant correlation between oil prices and stock returns in the automotive industry between November 2000 and December 2002, as well as between March 2006 and December 2009. Further, In Long-term co-movement was more pronounced and increased oil prices had an impact on stock return. A symmetric and asymmetric model was used by Bhatia, P. and Gupta, P. (2020) to study the volatility of Indian banking sectoral indices during the subprime crisis and COVID-19. During the crisis, there was a high level of volatility with a leverage effect; however the Nifty bank index as well as the private sector bank index disappeared during COVID-19. It suggests diversification as a strategy for long-term investors. With an emphasis on the Markowitz portfolio allocation, the global minimum variance portfolio problem, and the maximum return portfolio allocation, Trabelsi *et al. *(2021) investigated the correlation between gold returns and seven BSE sectoral indexes. The findings demonstrate that gold returns are unaffected by BSE indices and that gold returns may be used to forecast future returns for the Consumer Durables, Fast-Moving Consumer Goods, and Oil & Gas stock indexes. At the sectoral level in India, Mishra, S. and Mishra, S. (2021) examined the impact of disentangled oil shocks, including supply, demand, and risk-driven shocks. According to Markov regime regression, demand shocks benefit every sector in both high- and low-volatility regimes, suggesting that the Indian industry has a high level of consumption demand. Shocks to the supply are negligible in both high- and low-volatility environments. All sectors indicators are impacted by supply and demand shocks, both positively and negatively. Risk shocks affect all sectoral indexes negatively and statistically significantly. Al-hajj *et al. *(2021) evaluated the association between oil price shocks and stock market returns across nine economic sectors listed on Bursa Malaysia. Long-term ARDL correlations revealed that oil price shocks have a large negative influence on stock market returns in the property, mining, and technology sectors. On the other hand, interest rates and industrial production had a substantial positive influence on the returns of most sectors, but the exchange rate and inflation had a big negative impact on the stock market returns of the majority of sectors. Pandey (2022) examined the dynamic linkages between the movement of Indian stock market sectoral indices and the three macroeconomic variables and found that oil price, gold price and exchange rate simultaneously have a significant effect on sectoral indices in Indian stock market. The exchange rate has a significant negative impact on all sectors. Hashmi *et al. *(2022) used VAR-DCC-GARCH model to studied impact of Brent crude oil on Chinese stock market and selected industries and found that Shanghai Composite Index and selected industries are significantly influenced by Brent crude oil. Steel, nonferrous metals, chemical and mining industries are substantially affected. Ali *et al. *(2023) investigates the relationship between sectoral indices returns and inflation using the Pearson correlation method and found that all sectoral indices have a statistically significant relationship with inflation except Energy Index, Metal Index and IT Index.

This study attempts to fill the following gap in the existing literature by analyzing effect of Crude Oil Price (COP), Economic Policy Uncertainty Index (EPU), Exchange Rate (ER), Foreign Institutional Investment (FII), Wholesale Price Index (WPI), Index of Industrial Production (IIP) and Money Supply (MS) on selected sectoral indices of NSE.

First, previous studies have focused on effect of macroeconomic variables on aggregate stock market and have identified a significant effect on stock market ( Pethe & Karnik, 2000; Venkatraja, B., 2014; Muthukumar *et al. *, 2024 and Hedau, A., 2024). Changes in macroeconomic factors may have varying effects on various sectors. There are scant studies on effect of macroeconomic variables on sectoral indices which will be beneficial for investors to optimize their portfolio.

Second, Mishra, S., and Mishra, S. (2021), Al-hajj *et al. *(2021), Hashmi *et al. *(2022) and Mandal, K. and Datta, R. P. (2024) have studied effect of crude oil price on sectoral indices and found significant effect on sectoral indices, but these studies are limited to Effect of crude oil price.

## Method

In this study monthly data from April 2012 to August 2024 is used to analyze the influence of Crude Oil Price (COP), Economic Policy Uncertainty Index (EPU), Exchange Rate (ER), Foreign Institutional Investment (FII), Wholesale Price Index (WPI), Index of Industrial Production (IIP) and Money Supply (MS) on return of Nifty Auto, Nifty Bank, Nifty Financial Services, Nifty FMCG, Nifty IT, Nifty Media, Nifty Metal, Nifty Pharma and Nifty Realty ( Table 1).

**Figure 1.**
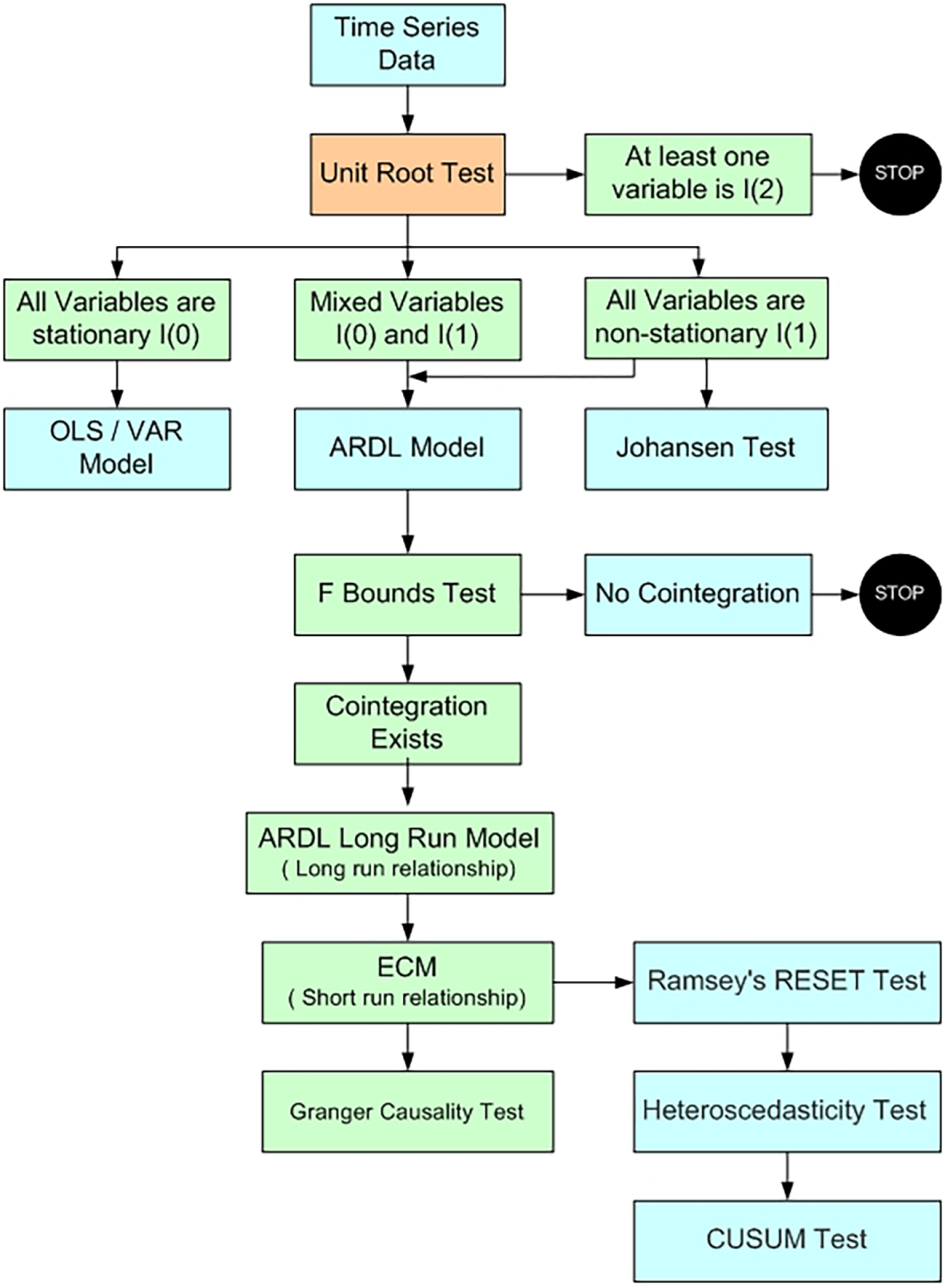
Model selection ( Singh Chauhan *et al.*, 2024).

**Table 1.** Variables of the study.

S.No	Variables	Symbol	Data source	Unit of measurement
1	Nifty Auto	LnAuto	Investing.com	Return
2	Nifty Bank	LnBank	Investing.com	Return
3	Nifty Financial Services	LnFS	Investing.com	Return
4	Nifty FMCG	LnFMCG	Investing.com	Return
5	Nifty IT	LnIT	Investing.com	Return
6	Nifty Media	LnMedia	Investing.com	Return
7	Nifty Metal	LnMetal	Investing.com	Return
8	Nifty Pharma	LnPharma	Investing.com	Return
9	Nifty Realty	LnRealty	Investing.com	Return
10	Crude Oil Price	LnCOP	Petroleum Planning & Analysis Cell	USD/Barrel
11	Economic Policy Uncertainty Index	LnEPU	FRED	Index
12	Exchange Rate	LnER	Investing.com	INR/USD
13	Foreign Institutional Investment	LnFII	Central Depository Services (India) Limited	INR (Cr)
14	Wholesale Price Index	LnWPI	Office of The Economic Adviser	Index
15	Index of Industrial Production	LnIIP	MOSPI	Index
16	Money Supply (M3)	LnMS	RBI	INR

The current study applied Pesaran *et al.* (2001) Autoregressive Distributed Lag (ARDL) model. This technique is utilized as it offer robust results when the dataset is limited in size and the variables exhibit a mix of integration orders, i.e., a combination of I(0) and I(1) variables. Before applying the ARDL model we need to check that none of the variables are stationery at second difference. The Augmented Dickey-Fuller Phillips, P. C. B. (1988) is employed to check at which level variables are stationery.

Equation of the augmented Dickey-Fuller test is as follows (Equation 1)

$$y_{t}=\alpha +\beta_{t}+\gamma y_{t-1}+\sum_{i=1}^{p}\delta_{i}∆y_{t-i}+e_{t}$$ (1)

In ADF test following hypotheses are tested:

H0: Series has a unit root

H1: Series has no unit root

Results of ADF test ( Table 3) support application of ARDL to examine the cointegration between the variables. If the variables are stationery at level Johansen cointegration test is suitable to study the long term relationship among the variables.

Equation expressing the ARDL model specification is as follows (Equation 2)

$$∆{\text{LnNSE}}_{t}^{k}=\alpha_{0}+\sum_{i-1}^{p}\alpha_{1}∆{\text{LnNSE}}_{t-1}^{k}+\sum_{i-1}^{q1}\alpha_{2}∆{\text{LnCOP}}_{t-1}+\sum_{i-1}^{q2}\alpha_{3}∆{\text{LnEPU}}_{t-1}+\sum_{i-1}^{q3}\alpha_{4}∆{\text{LnER}}_{t-1}+\sum_{i-1}^{q4}\alpha_{5}∆{\text{LnFII}}_{t-1}+\sum_{i-1}^{q5}\alpha_{6}∆{\text{LnWPI}}_{t-1}+\sum_{i-1}^{q6}\alpha_{7}∆{\text{LnIIP}}_{t-1}+\sum_{i-1}^{q7}\alpha_{8}∆{\text{LnMS}}_{t-1}+\delta_{1}{\text{LnNSE}}_{t-1}^{k}+\delta_{2}{\text{LnCOP}}_{t-1}+\delta_{3}{\text{LnEPU}}_{t-1}+\delta_{4}{\text{LnER}}_{t-1}+\delta_{5}{\text{LnFII}}_{t-1}+\delta_{6}{\text{LnWPI}}_{t-1}+\delta_{7}{\text{LnIIP}}_{t-1}+\delta_{8}{\text{LnMS}}_{t-1}+\varepsilon_{t}$$ (2)

In the above equation k = Nifty Auto, Nifty Bank, Nifty Financial Services, Nifty FMCG, Nifty IT, Nifty Media, Nifty Metal, Nifty Pharma and Nifty Realty. Δ is the first difference operator; α represent short-term relationship; δ represent long-term relationship; p and q represent restricted lags and $\varepsilon_{t}$ shows error term.

The variables are said to be cointegrated if the calculated F-statistic is greater than the upper bound critical value, suggesting a long-term link between them. On the other hand, the test is considered inconclusive if the F-statistic lies between the lower bound critical value and the upper bound critical value. It is concluded that the variables are not cointegrated when the F-statistic is smaller than the lower bound critical value. Upon confirmation of cointegration by the ARDL bounds test, the subsequent step involves estimating the long-run coefficients and short-run parameters, including the error correction term ( Shrestha, M. B., & Bhatta, G. R. 2018). Finally diagnostic tests are use to check the reliability of the model. Ramsey’s RESET test assesses the functional form of the model, the Breusch–Pagan–Godfrey test evaluates heteroscedasticity, the Breusch–Godfrey serial correlation Lagrange multiplier (LM) test examines serial correlation among the residuals, and the CUSUM test analyzes the stability of coefficients.

## Results and Discussion

Descriptive Statistics of the variables is presented in Table 2. Natural log value of the variables in the study is used for analysis. The results indicate that the mean of money supply is highest (16.6252) and mean of Nifty Media is lowest (0.0036). Moreover, the standard deviation indicates that FII is most volatile as compared to other variables in the study. FII has the highest Kurtosis among all the variables. Nifty FMCG, Nifty PHARMA, EPU, MS and WPI are positively skewed whereas rest of the variables are negatively skewed.

**Table 2.** Descriptive statistics.

	Mean	Maximum	Minimum	Std. Dev.	Skewness	Kurtosis	Jarque-Bera	P value
** LNAUTO**	0.0122	0.2210	-0.3779	0.0677	-1.4255	9.8861	344.8495*	0.0000
**LNBANK**	0.0108	0.2142	-0.4204	0.0723	-1.2074	10.5972	394.5277*	0.0000
**LNFMCG**	0.0115	0.1205	-0.1007	0.0409	0.1187	2.6631	1.0543	0.5903
**LNFS**	0.0117	0.2057	-0.3757	0.0651	-1.2385	10.5269	389.8176*	0.0000
**LNMEDIA**	0.0036	0.2890	-0.4741	0.0808	-0.9786	10.8680	408.1112*	0.0000
**LNMETAL**	0.0075	0.2219	-0.3480	0.0859	-0.3396	4.4107	15.2181*	0.0005
**LNIT**	0.0126	0.2028	-0.1770	0.0606	-0.1572	3.7274	3.8983	0.1424
**LNPHARMA**	0.0102	0.2621	-0.1362	0.0545	0.4822	5.1669	34.9248*	0.0000
**LNREALTY**	0.0098	0.3044	-0.4691	0.1012	-0.4905	5.8249	55.5176*	0.0000
**LNCOP**	4.2394	4.7700	2.9900	0.3537	-0.5822	3.0364	8.4258	0.0148
**LNEPU**	4.4022	5.6479	3.1507	0.4459	0.0107	2.8331	0.1758	0.9159
**LNER**	4.2318	4.4293	3.9640	0.1227	-0.1753	2.3630	3.2820	0.1938
**LNFII**	11.6524	12.2197	0.0000	0.9805	-11.3721	135.6720	112489.8000*	0.0000
**LNIIP**	4.8101	5.0752	3.9890	0.1371	-1.4213	10.0494	358.6770*	0.0000
**LNMS**	16.6252	17.7587	15.8346	0.5607	0.5750	2.1493	12.7039*	0.0017
**LNWPI**	4.8138	5.0460	4.6511	0.1247	0.7649	2.1050	19.5036*	0.0001

In time series analysis stationarity of the variables is checked to select the test to be applied for analysis. Augmented Dickey-Fuller test to is applied to ascertain stationarity of the variables. The results of ADF test ( Table 3) show that all the sectoral indices are stationary at level I(0) and among macroeconomic variables LnEPU and LnFII are stationary at level I(0) whereas LnCOP, LnER, LnWPI, LnIIP and LnMS are stationary at first difference I(1). Upon the initial differencing, all non-stationary series attain stationarity.

**Table 3.** Unit root test (ADF).

Null Hypothesis: Series has a unit root					
	Level		First difference		Order of Integration
	t-Statistic	p-Value	t-Statistic	p-Value	
**LnAuto**	-11.6584	0.0000			I(0)
**LnBank**	-12.7356	0.0000			I(0)
**LnFS**	-12.5840	0.0000			I(0)
**LnFMCG**	-12.6787	0.0000			I(0)
**LnIT**	-12.6260	0.0000			I(0)
**LnMedia**	-12.4558	0.0000			I(0)
**LnMetal**	-10.7459	0.0000			I(0)
**LnPharma**	-12.7520	0.0000			I(0)
**LnRealty**	-11.2989	0.0000			I(0)
**LnCOP**	-2.6758	0.0807	-9.1471	0.0000	I(1)
**LnEPU**	-6.0284	0.0000			I(0)
**LnER**	-1.5252	0.5182	-13.4417	0.0000	I(1)
**LnFII**	-11.7821	0.0000			I(0)
**LnWPI**	-0.1838	0.9366	-6.6802	0.0000	I(1)
**LnIIP**	-3.3126	0.0160	-17.0053	0.0000	I(1)
**LnMS**	-1.3157	0.6214	-13.2850	0.0000	I(1)

Result of ADF test reveal that some variables are stationary at level I(0) and some are stationary at first difference I(1). When variables are mixture of I(0) and I(1) the most appropriate statistical technique to study relationship among variables is ARDL approach. So to study relationship among the variables ARDL approach is employed in the study.

Result of the Autoregressive Distributed Lag Bounds Test ( Table 4) indicates a significant long run relationship among selected macroeconomic variables and sectoral indices of NSE. The calculated F-statistic of all sectoral indices exceeds the upper bound critical value at 1% level of significance Pesaran *et al.* (2001). Consequently, the null hypothesis is rejected, signifying that the dependent variable exhibits a long-run relationship with the independent variables.

**Table 4.** ARDL bounds test.

	F-statistic	I(0)	I(1)
**LnAuto**	16.5361	2.73	3.90
**LnBank**	10.3679	2.73	3.90
**LnFS**	9.7011	2.73	3.90
**LnFMCG**	12.7684	2.73	3.90
**LnIT**	20.8701	2.73	3.90
**LnMedia**	10.9839	2.73	3.90
**LnMetal**	21.8664	2.73	3.90
**LnPharma**	27.4475	2.73	3.90
**LnRealty**	12.5666	2.73	3.90

Bound critical values at 1% level of significance.

Once the long term relationship between selected macroeconomic variables and sectoral indices is identified, the next step is to check how the selected macroeconomic variables influence the sectoral indices of NSE. Long run Coefficient of the selected macroeconomic variables with respect to sectoral indices of NSE is presented in Table 5.

**Table 5.** Long run form of ARDL.

Variables	LnAuto	LnBank	LnFS	LnFMCG	LnIT	LnMedia	LnMetal	LnPharma	LnRealty
** LnCOP**	-0.0426	0.0094	0.0097	-0.027	0.0215	-0.0529**	-0.0422	-0.0035	0.0059
	[-1.6037]	[0.4549]	[0.5060]	[-1.6294]	[0.8283]	[-2.2635]	[-1.0143]	[-0.1696]	[0.1839]
**LnEPU**	0.0286**	0.0004	0.0004	0.0199**	0.0128	-0.0063	0.0070	0.0173***	-0.0040
	[2.4153]	[0.0434]	[0.0480]	[2.4234]	[1.1266]	[-0.6518]	[0.4201]	[1.9595]	[-0.3204]
**LnER**	-0.1009	0.1461	0.1268	-0.1388	0.1753	-0.1971	0.2044	0.0375	0.0020
	[-0.6341]	[1.1880]	[1.1037]	[-1.5217]	[1.1502]	[-1.4841]	[0.8888]	[0.3161]	[0.0102]
**LnFII**	0.0368*	0.0341*	0.0300*	0.0055***	0.0235	0.0486*	0.0264*	0.0102*	0.0281*
	[2.6260]	[3.8907]	[3.6561]	[1.9692]	[1.4616]	[4.5128]	[3.6568]	[2.8645]	[4.7885]
**LnIIP**	-0.2050**	-0.0810	-0.0669	0.0415	-0.1692***	-0.3136*	-0.2289*	-0.1890*	-0.0459
	[-2.3764]	[-1.4306]	[-1.2748]	[0.9705]	[-1.8051]	[-4.1919]	[-2.9020]	[-4.7459]	[-0.7441]
**LnMS**	-0.0006	-0.0239**	-0.0156	-0.0150***	0.0278***	0.0033	-0.0231	0.0106	0.0221
	[-0.0446]	[-2.0666]	[-1.4378]	[-1.6782]	[1.8035]	[0.2557]	[-1.0385]	[0.8695]	[1.3066]
**LnWIP**	0.3255**	0.0136	-0.0168	0.1766***	-0.1403	0.4007*	0.1678	0.1077	0.0458
	[2.1542]	[0.1177]	[-0.1553]	[1.8615]	[-0.9179]	[3.0059]	[0.7117]	[0.8871]	[0.2511]
**Constant**	-0.5006**	-0.3206**	-0.2509	-0.2354***	-0.1219	0.0500	-0.3351	-0.1147	-0.6880*
	[-2.4426]	[-2.1638]	[-1.8087]	[-1.7913]	[-0.5320]	[0.2764]	[-1.1343]	[-0.7111]	[-3.1534]
**Model**	(1, 2, 1, 4, 4, 2, 0, 4)	(3, 0, 0, 4, 2, 0, 0, 3)	(3, 0, 0, 4, 2, 0, 0, 3)	(2, 4, 1, 1, 0, 4, 0, 0)	(1, 0, 0, 0, 4, 1, 0, 0)	(3, 4, 0, 1, 2, 1, 0, 0)	(1, 3, 0, 1, 0, 0, 0, 0)	(1, 0, 0, 0, 0, 0, 0, 0)	(4, 0, 0, 4, 0, 0, 0, 0)

Note: *, ** and *** Indicate significance at the 1%, 5% and 10% level, respectively.

Table 5 indicates that effect of crude oil price (COP) on LnMedia is statistically significant and negative, which is consistent with the acceptable concept that increase in crude oil price increases cost of the companies and as a result negatively affect their profit margin and share price. This outcome is consistent with the result of studies conducted by Chancharat *et al. *(2007), Basher *et al. *(2012) and Arfaoui, M. and Ben Rejeb, A. (2017). However, the influence of crude oil price is statistically insignificant in the case of LnAuto, LnBank, LnFS, LnFMCG, LnIT, LnMetal, LnPharma and LnRealty. It shows that crude oil price is not a significant determinant for share price of these sectors. Similar findings were observed in the studies conducted by Henriques, I. and Sadorsky, P. (2008), Cong *et al. *(2008), Jammazi, R., and Aloui, C. (2010) and Raju *et al.* (2021).

Economic conditions in a country can have a substantial impact on the stock market. Resilient stock markets require robust economic conditions, political stability, rule of law, and effective governance structures. Without these conditions, the stock market is vulnerable to external shocks. Prosperity or misery of a country’s economic fate is largely dictated by the political condition existing in a nation Bilson *et al.* (2002). Economic Policy Uncertainty Index developed by Baker *et al. *(2016) is one if the index to measure the policy-related economic uncertainty. The effect Economic Policy Uncertainty Index is positive and statistically significant on LnAuto, LnFMCG and LnPharma ( Table 5). This may be due to various economic reforms in India like Implementation of GST, demonetization, make in India initiative during the study period. These reforms initially caused uncertainty but in the long run these were perceived to be growth drivers, leading to positive effect on stock market. However, the influence of Economic Policy Uncertainty Index is statistically insignificant in the case of LnBank, LnFS, LnIT, LnMedia, LnMetal and LnRealty.

Long run dynamics ( Table 5) indicates that effect of Exchange rate is statistically insignificant for all the sectoral indices analysed in the study. Therefore, it can be said that exchange rate is not relevant for prediction of stock market return. The finding is consistent with the study conducted by Gan *et al. *(2006), Oseni, I. O. and Nwosa, P. I. (2011), Raju *et al. *(2021) and Syed, A. A. (2021).

Foreign institutional investment is one of the most significant variables influencing stock price movement, particularly in the context of growing economies such as India. Due to their higher returns than those of other international stock markets, Indian stock markets have been able to draw a significant amount of foreign institutional investment since September 1992, when the investing opportunity was opened to foreign institutional investors ( Bhargava & Malhotra, 2015). High amount of inflow and outflow may increase the volatility in the stock market. This volatility may destabilize retail investors and create uncertainty in the broader financial system. It becomes important to analyze the effect of FII on the stock market, so that investors can take decision related to their investments and minimize the risk of loss. Analysis in Table 5 indicates that effect of FII is statistically significant and positive on LnAuto, LnBank, LnFS, LnFMCG, LnMedia, LnMetal, LnPharma and LnRealty, however effect on LnIT is statistically insignificant. This indicates that FII is the important determinant of stock return for all the selected sectors. The results are in line with finding of the studies conducted by Dhingra *et al.* (2016), Sultana, S. T. and Reddy, K. S. (2017) and Rao, V. and Jyotreddy, K. (2022). Venkatraja, B. (2014) also concluded that BSE sensex in significantly affected by foreign institutional investments FII.

IIP is a measure of the economy’s actual level of activity. An increase in IIP is anticipated to have a favorable impact on cash flows and earnings, which will subsequently have a positive effect on the stock price ( Mukherjee & Naka, 1995). The ARDL result presented in Table 5 indicate that effect of IIP is negative and statistically significant on LnAuto, LnIT, LnMedia, LnMetal, LnPharma. This outcome is consistent with the studies conducted by Bhattacharjee, A. and Das, J. (2020) and Kaur, P. and Singla, R. (2021). However this outcome is inconsistent with studies conducted by Humpe, A., and Macmillan, P. (2009), Maji, S. K., Laha, A. and Sur, D. (2020) and Pole, H. and Cavusoglu, B. (2021). The influence of IIP is insignificant on LnBank, LnFS, LnFMCG and LnRealty.

The results shown in Table 5 indicate that effect of money supply (M3) is positive and statistically significant on LnIT. This finding is consisted with the outcome of studies conducted by Olowe, R. A. (2007), Sahu, T. N. and Pandey, K. D. (2020) and Bhattacharjee, A. and Das, J. (2021). However, the effect is negative and statistically significant on LnBank and LnFMCG. This finding is consistent with the studies conducted by Rjoub, H., Türsoy, T. and Günsel, N. (2009), Alam, Z., and Rashıd, K. (2014) and Asravor, R. K. and Fonu, P. D. D. (2021). The effect is insignificant in case of LnAuto, LnFS, LnMedia, LnMetal, LnPharma and LnRealty.

Evidence from empirical studies has shown that inflation have a positive influence on stock prices. According to Marshall (1992), during inflation, the demand for money declines along with the anticipated return on money and the stock will be positively affected from an increase in the demand for equity. Analysis shown in Table 5 indicates that the effect of WPI is positive and statistically significant on LnAuto, LnFMCG and LnMedia. In Table 5 coefficients associated with WPI are positive for all the sectoral indices except LnFS and LnIT. Thus, it can be said sectoral indices of NSE are positively affected by inflation. This outcome is consistent with the studies conducted by Olowe, R. A. (2007), Ratanapakorn, O. and Sharma, S. C. (2007), Tripathi, V. and Seth, R. (2014) and Bhattacharjee, A. and Das, J. (2020).

Table 6 shows the short run dynamics and equilibrium relationship between selected macroeconomic variables and sectoral indices of NSE. A relationship may be stable over the long run, but there may be disequilibrium in the short run. It is essential that the error correction coefficient is statistically significant and negative in order to establish long-run equilibrium. The results of ECM show that error correction coefficient for all the sectoral indices are significant and negative. This supported the earlier claim that selected macroeconomic variables and sectoral indices have a long-run equilibrium relationship Maji *et al*. (2020), Al-hajj *et al*. (2021) and Sharma, A. K. (2024). It also implies that disequilibrium from the long run is rectified in the next month at a high speed. Furthermore, the model demonstrates strong explanatory power, with a high value of R-squared for all the sectors, highlighting its robustness in capturing the interrelationships among the variables.

**Table 6.** Short run relationship.

Variables	LnAuto	LnBank	LnFS	LnFMCG	LnIT	LnMedia	LnMetal	LnPharma	LnRealty
DLnRealty (t-1)									0.2383 [2.2653]**
DLnRealty (t-2)									0.2650 [3.0377]*
DLnRealty (t-3)									0.1122 [1.8346]***
DLnMedia (t-1)						0.3248 [2.8829]*			
DLnMedia (t-2)						0.1704 [2.5429]**			
DLnFS (t-1)			0.3266 [2.8610]*						
DLnFS (t-2)			0.1663 [2.4894]**						
DLnFMCG (t-1)				0.1664 [2.2984]**					
DLnBank (t-1)		0.3398 [2.9676]*							
DLnBank (t-2)		0.1534 [2.2934]**							
DLnCOP (t)	0.0527 [1.0044]			-0.0521 [-1.6500]		0.0056 [0.0861]	0.0857 [1.4137]		
DLnCOP (t-1)	0.0972 [1.6761]***			0.0720 [2.0203]**		0.0689 [1.1507]	0.1314 [2.1555]**		
DLnCOP (t-2)				0.0176 [0.4829]		0.0790 [1.4389]	0.0961 [1.5960]		
DLnCOP (t-3)				0.0722 [2.1128]**		0.1238 [2.4277]**			
DLnEPU (t)	0.0052 [0.5227]			0.0063 [0.8242]					
DLnER (t)	-1.1191 [-5.2818]*	-1.5379 [-6.5589]*	-1.4079 [-6.7078]*	-0.8389 [-5.0258]*		-1.2862 [-4.3646]*	-0.9312 [-2.7724]*		-2.5265 [-7.1797]*
DLnER (t-1)	-0.1734 [-0.7252]	-0.6382 [-2.3478]**	-0.4530 [-1.8529]***						-1.4031 [-3.4232]*
DLnER (t-2)	0.5305 [2.4332]**	-0.4038 [-1.4361]	-0.2634 [-1.0513]						-0.3016 [-0.7322]
DLnER (t-3)	-0.3148 [-1.5160]	-0.6290 [-2.3207]**	-0.6077 [-2.5091]**						-1.0667 [-2.7045]*
DLnFII (t)	0.035 [8.8308]*	0.03434 [9.5871]*	0.0308 [9.6675]*		0.0192 [4.6441]*	0.0374 [7.5465]*			
DLnFII (t-1)	0.0131 [1.5689]	-0.0144 [-3.3085]*	-0.0137 [-3.4697]*		-0.0100 [-1.3350]	-0.0241 [-2.7263]*			
DLnFII (t-2)	0.0217 [2.5793]**				0.0081 [1.5348]				
DLnFII (t-3)	0.011 [2.6372]*				0.0117 [2.8390]*				
DLnIIP (t)	-0.4968 [-5.8803]*			-0.0207 [-0.5449]	0.0150 [0.1896]	-0.1992 [-2.2504]**			
DLnIIP (t-1)	-0.2773 [-3.3741]*			-0.1382 [-3.1920]*					
DLnIIP (t-2)				-0.1025 [-2.3737]**					
DLnIIP (t-3)				-0.1374 [-3.5222]*					
DLnWPI (t)	0.614 [0.8738]	0.9385 [1.5493]	0.8427 [1.5594]						
DLnWPI (t-1)	-1.9723 [-2.7173]*	-0.9790 [-1.3564]	-0.9011 [-1.3951]						
DLnWPI (t-2)	2.5783 [3.7473]*	1.3513 [2.2300]**	1.2026 [2.2196]**						
DLnWPI (t-3)	-0.8519 [-1.5545]								
CointEq(-1)*	-1.0475 [-12.6028]*	-1.4354 [-9.9640]*	-1.3651 [-9.6384]*	-1.2106 [-11.0576]*	-1.1070 [-14.1174]*	-1.5191 [-10.2534]*	-1.0052 [-14.4442]*	-1.18251 [-16.1631]*	-1.4046 [-10.9596]*
R-squared	0.8005	0.7984	0.8022	0.6749	0.6329	0.7484	0.6122	0.6399	0.7222
Adjusted R-squared	0.7738	0.7818	0.7859	0.6481	0.6197	0.7296	0.6012	0.6399	0.7080
Durbin-Watson stat	1.9550	1.9919	1.9919	2.0648	1.9437	2.0257	1.9881	1.9416	2.0869
F-statistic	16.5361	10.3679	9.7011	12.7684	20.8701	10.9839	21.8664	27.4475	12.5666

Note: *, ** and *** Indicate significance at the 1%, 5% and 10% level, respectively.

**Figure 2.**
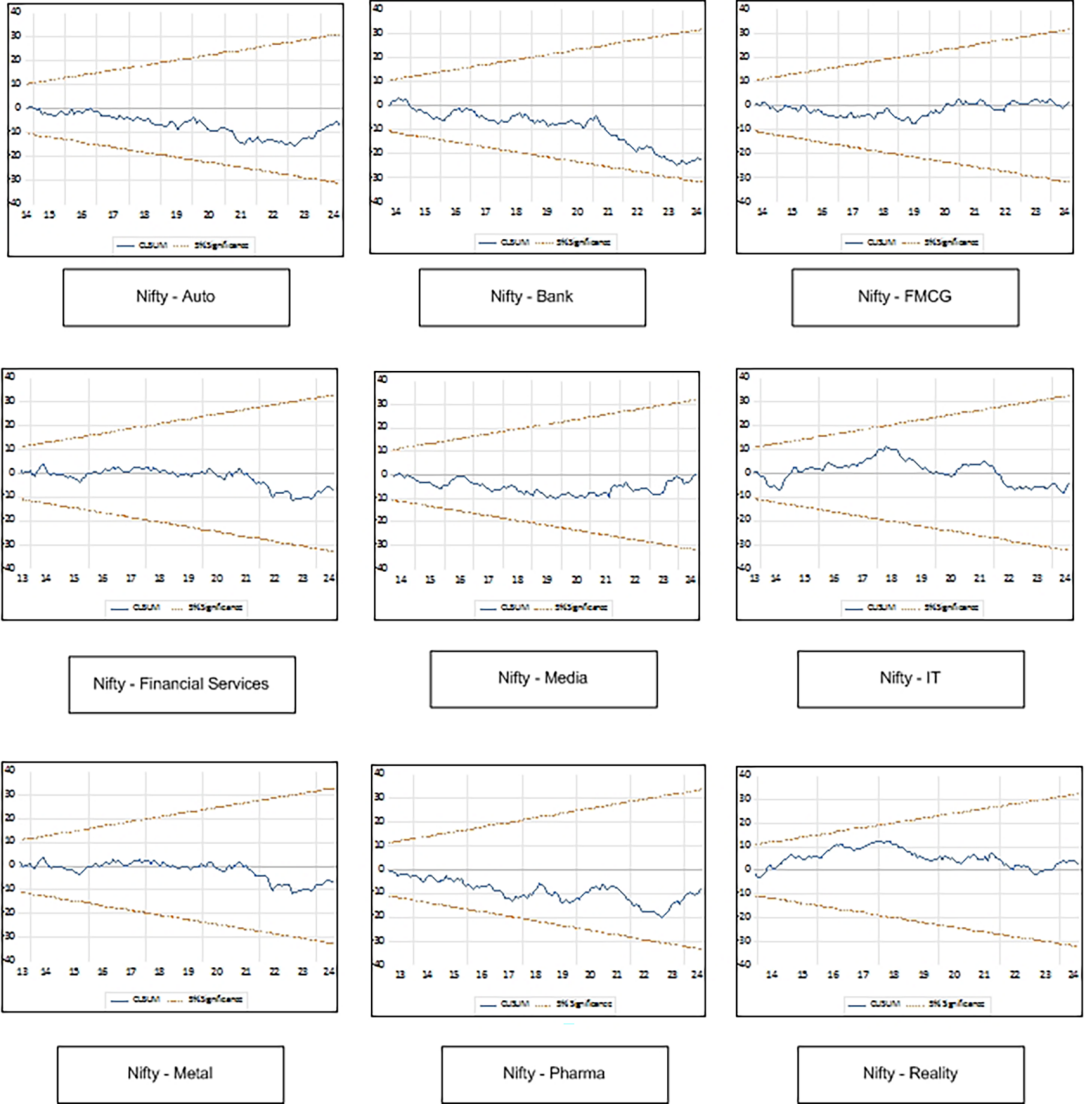
CUSUM plot.

Results of diagnostic tests applied to check reliability of the model is presented in Table 7. The Ramsey RESET test indicates no evidence of model misspecification for LnAuto, LnFMCG, LnIT, LnMedia, LnMetal and LnPharma. Heteroskedasticity Test indicates no heteroskedasticity for all the sectors and no serial correlation is reported by Breusch-Godfrey Serial Correlation LM Test. CUSUM plot presented in Figure 2 indicate that the model is stable and outcomes are reliable as line for all the sector indices is well within the upper and lower bounds of 5 percent level.

**Table 7.** Diagnostic test results.

Diagnostic test	LnAuto	LnBank	LnFS	LnFMCG	LnIT	LnMedia	LnMetal	LnPharma	LnRealty
Ramsey RESET Test, F-statistic	0.1129	12.3052	15.7301	0.1952	2.5300	3.0986	1.9549	1.4152	11.3821
	[0.7375]	[0.0006]	[0.0001]	[0.6594]	[0.1141]	[0.0808]	[0.1644]	[0.2362]	[0.0010]
Heteroskedasticity Test: Breusch-Pagan-Godfrey, F-statistic	0.6188	0.9962	0.8120	0.9376	1.7613	1.1815	1.2728	0.4740	0.8746
	[0.9175]	[0.4701]	[0.6894]	[0.5386]	[0.0559]	[0.2861]	[0.2419]	[0.8729]	[0.5936]
Breusch-Godfrey Serial Correlation LM Test:	1.0073	0.3957	0.8155	0.9570	0.3882	0.1381	0.5290	0.1888	1.5376
	[0.3684]	[0.6741]	[0.4448]	[0.3869]	[0.6791]	[0.8712]	[0.5904]	[0.8281]	[0.2189]

Note: Test statistics (P-Value mentioned in bracket).

## Conclusion and Recommendations

In this study, we have analyzed the influence of Crude Oil Price (COP), Economic Policy Uncertainty Index (EPU), Exchange Rate (ER), Foreign Institutional Investment (FII), Wholesale Price Index (WPI), Index of Industrial Production (IIP) and Money Supply (MS) on return of Nifty Auto, Nifty Bank, Nifty Financial Services, Nifty FMCG, Nifty IT, Nifty Media, Nifty Metal, Nifty Pharma and Nifty Realty. ADF test indicates that some variables are stationary at level I(0) and some are stationary at first difference I(1). This necessitates use of ARDL model to study relationship between the variables. ARDL bond test indicates a significant long run relationship among selected macroeconomic variables and sectoral indices of NSE. The analysis of long run relationship shows that Foreign Institutional Investment (FII) significantly affects all the sectoral indices except IT. Index of industrial production (IIP) have significant relationship with Auto, IT, Media, Metal and Pharma. Money supply (MS) significantly affects Bank, FMCG and IT in the long run. Wholesale Price Index (WPI) has significant relationship with Auto, FMCG and Media in the long run. Economic Policy Uncertainty Index affects Auto, FMCG and Pharma in the long run. Crude oil price has significant effect only on Media in the long run. Exchange rate does not have significant effect on any of the sectoral index. So, in the long run FII, IIP, EPU, MS and WIP are major determinants of stock market return. In the short run FII, ER and COP are major determinants of stock market return. Moreover, the result of diagnostic tests show that no model misspecification, no heteroskedasticity and no serial correlation. CUSUM plot indicate that the model is stable and outcomes are reliable.

Policy implications of the study are: Firstly, the findings are expected to be beneficial to the researchers and policymakers in understanding the relationship between selected macroeconomic variables and stock market return concerning different sectors in NSE. Secondly, the finding will enable the portfolio managers and investors to make prudent investment decision making concerning different sectors based on the change in the macroeconomic indicators as the response of stock belonging to various sectors to change in macroeconomic indicator precisely determined in the short run and long run.

## Ethics and consent

Ethical approval and consent were not required.

## Data Availability

**Figshare:** Exploring the Relationship between Macroeconomic Indicators and Sectoral Indices of Indian Stock Market. https://doi.org/10.6084/m9.figshare.28123442 ( Chauhan *et al.*, 2025). This project contains the following underlying data:
• Exploring the Relationship between Macroeconomic Indicators on Sectoral Indices of Indian Stock Market.xlsx Exploring the Relationship between Macroeconomic Indicators and Sectoral Indices of Indian Stock Market © 2025 by Sanjay Singh Chauhan, Dr. Pradeep Suri, Dr. Bhekisipho Twala, Dr. Neeraj Priyadarshi, Dr. Farman Ali is licensed under CC BY 4.0
