# Supplementary material for: Exploring the relationship between macroeconomic indicators and sectoral indices of Indian stock market
Source: F1000Res. 2025 Apr 7;14:180. Originally published 2025 Feb 10. [Version 2] doi: 10.12688/f1000research.160668.2 (PMC12001019; doi:10.12688/f1000research.160668.2)
Supplement: Peer review history

# Reviewer report: Reviewer response for version 2

**DOI:** 10.5256/f1000research.180078.r377139

**Published:** 2025-04-29

**Authors:** Dipendra Karki

**Affiliations:** 1 Nepal Commerce Campus, Tribhuvan University, Kirtipur, Central Development Region, Nepal

**Review Report**

**Overview**

 The manuscript presents an empirical exploration into the dynamic relationships between key macroeconomic indicators and sectoral stock indices of the Indian stock market over the period April 2012 to august 2024. Applying the ARDL method, the study reveals both short-run and long-run relationships, with notable findings such as FII’s significant impact on most sectors. The work is methodologically sound, addresses a relevant research question. However, certain improvements are required before the manuscript can be considered for publication to enhance its clarity and contribution. Some of the areas for improvement is mentioned below.

**Abstract:**

 The abstract clearly summarizes the purpose, methods, and key findings of the study. However,

- the limitations of the study should be explicitly mentioned to guide future research directions.
- Avoid abbreviations in the abstract to ensure broader accessibility for readers unfamiliar with technical terms.
- Include implications and recommendations for target groups like academia, practitioners, and policymakers.

**Introduction:**

 The introduction provides a reasonable background but needs more precise identification of the core research issues and problems the study seeks to address.

- The final paragraph of the Introduction should briefly outline the organization of the study, describing how subsequent sections are arranged (e.g., literature review, methodology, results, discussion, conclusion).

**Literature Review:**

 The manuscript reviews relevant literature but needs to incorporate more recent studies especially after 2020 to reflect updated trends and developments in the field.

- The research gap is mentioned, but insufficiently developed. Strengthen it by including:
  - *Contextual gaps* (specific to the Indian stock market or sectoral studies),
  - *Methodological gaps* (such as the use of ARDL compared to alternative approaches),
  - *Evidence-based gaps* (e.g., inconsistency of macroeconomic variables' impact across sectors).

**Methods:**

 The justification for using the ARDL model is well-established, particularly based on mixed orders of integration (I(0) and I(1)) shown in the ADF test (Table 3). ARDL is appropriate for small sample sizes and mixed stationary data, which reinforces its methodological suitability.

- *Optimal lag length selection criteria* should be reported clearly (such as AIC, SIC, or HQIC) before proceeding to the bounds testing and estimation stages.

**Results and Discussion:**

 The Bound Test (Table 4) correctly identifies long-run relationships, strengthening the study's findings.

- It is recommended to examine the influence of multiple macroeconomic variables simultaneously for each sectoral index in the ARDL estimation (Table 5). This aligns better with the title and offers richer insights. The absence of a multiple-variable long-run analysis per sector is a major limitation that should be clearly acknowledged in the discussion.
- Lacking to show the Error Correction Model (ECM) results. It should be included to assess the speed of adjustment toward long-run equilibrium after a short-term shock.
- Discuss whether the findings support or contradict existing Indian and global studies, providing a richer context to the results.
- Some tables (especially Table 5) could be enhanced with better explanation and interpretation of key coefficients, e.g., FII being significant (0.0368*, 0.0341*, 0.0300*, 0.0055***, 0.0235, 0.0486*, 0.0264*, 0.0102*, 0.0281*) but the exchange rate being consistently insignificant across sectors.

**Conclusion:**

 The conclusion effectively summarizes major findings but would enrich from:

- Highlighting study limitations (e.g., sector-specific idiosyncrasies, macroeconomic volatility),
- Discussing future research directions (e.g., incorporating dynamic models like VECM),

**Reviewer’s Decision:**

 The manuscript is deemed suitable for publication after **minor revisions** on the suggested areas to meet the standards required for publishing in F1000Research

Is the work clearly and accurately presented and does it cite the current literature?

Yes

If applicable, is the statistical analysis and its interpretation appropriate?

Yes

Are all the source data underlying the results available to ensure full reproducibility?

Partly

Is the study design appropriate and is the work technically sound?

Yes

Are the conclusions drawn adequately supported by the results?

Yes

Are sufficient details of methods and analysis provided to allow replication by others?

Partly

Reviewer Expertise:

My area of specialization lies to Behavioral finance, Capital Market, Investment Strategies, Financial Economics and Econometrics.

I confirm that I have read this submission and believe that I have an appropriate level of expertise to confirm that it is of an acceptable scientific standard, however I have significant reservations, as outlined above.

# Reviewer report: Reviewer response for version 2

**DOI:** 10.5256/f1000research.180078.r376379

**Published:** 2025-04-14

**Authors:** Werner R Murhadi

**Affiliations:** 1 Universitas Surabaya, Surabaya, East Java, Indonesia

We thank you for the improvement efforts that have been made by the author well. We hope this article can provide enlightenment to those who read it.

Is the work clearly and accurately presented and does it cite the current literature?

Yes

If applicable, is the statistical analysis and its interpretation appropriate?

Yes

Are all the source data underlying the results available to ensure full reproducibility?

Yes

Is the study design appropriate and is the work technically sound?

Yes

Are the conclusions drawn adequately supported by the results?

Yes

Are sufficient details of methods and analysis provided to allow replication by others?

Yes

Reviewer Expertise:

Financial and strategic management

I confirm that I have read this submission and believe that I have an appropriate level of expertise to confirm that it is of an acceptable scientific standard.

# Reviewer report: Reviewer response for version 1

**DOI:** 10.5256/f1000research.176599.r367784

**Published:** 2025-03-27

**Authors:** Mushtaq Ahmad Darzi

**Affiliations:** 1 University of Kashmir, Jammu & Kashmir, India

**Full Report**

**Summary of the Article:**

**Title:**

**Exploring the Relationship between Macroeconomic Indicators and Sectoral Indices of the Indian Stock Market**

**Background and Research Motivation:**

 The performance of stock markets is significantly influenced by macroeconomic indicators. However, different sectors respond differently to changes in macroeconomic factors, making it important for investors, policymakers, and researchers to understand these sectoral variations. This study aims to investigate the short-run and long-run effects of key macroeconomic indicators on sectoral indices of the Indian stock market.

**Research Objectives:**

1. To analyse the impact of seven key macroeconomic indicators on sectoral stock indices in India.
2. To determine the short-term and long-term relationships between macroeconomic factors and stock market performance.
3. To provide insights for investors, regulators, and policymakers regarding sector-specific market behaviour.

**Macroeconomic Indicators Considered:**

 The study evaluates the impact of the following macroeconomic indicators on nine sectoral indices of the National Stock Exchange (NSE):

1. Crude Oil Price (COP)
2. Economic Policy Uncertainty Index (EPU)
3. Exchange Rate (ER)
4. Foreign Institutional Investment (FII)
5. Wholesale Price Index (WPI)
6. Index of Industrial Production (IIP)
7. Money Supply (MS)

**Sectoral Indices Analysed:**

 The research focuses on nine sectoral indices from April 2012 to August 2024:

1. Nifty Auto
2. Nifty Bank
3. Nifty Financial Services (FS)
4. Nifty FMCG
5. Nifty IT
6. Nifty Media
7. Nifty Metal
8. Nifty Pharma
9. Nifty Realty

**Methodology:**

- The study employs the Autoregressive Distributed Lag (ARDL) model, which is well-suited for time series data containing a mix of stationary (I(0)) and non-stationary (I(1)) variables.
- The Augmented Dickey-Fuller (ADF) test is used to determine the stationarity of the variables.
- The ARDL bounds test is applied to confirm the existence of a long-run relationship between macroeconomic indicators and sectoral indices.
- Error Correction Model (ECM) is used to assess short-run dynamics.

**Key Findings:**

1. **Long-Run Relationship (ARDL Model Results)**

- Foreign Institutional Investment (FII):

- Positively affects all sectoral indices except IT.
- This suggests that foreign investments play a crucial role in determining stock prices across most sectors.

- Index of Industrial Production (IIP):

- Significantly impacts Auto, IT, Media, Metal, and Pharma sectors.
- Indicates that industrial production levels influence capital-intensive and technology-driven industries.

- Money Supply (MS):

- Positively affects Bank, FMCG, and IT sectors in the long run.
- Suggests that liquidity conditions in the economy support these sectors.

- Wholesale Price Index (WPI):

- Positively influences Auto, FMCG, and Media sectors.
- Indicates that inflationary trends drive pricing power and profitability in these industries.

- Economic Policy Uncertainty (EPU):

- Positively affects Auto, FMCG, and Pharma sectors.
- This suggests that while uncertainty initially disrupts the market, certain sectors benefit from economic reforms and policy shifts over time.

- Crude Oil Price (COP):

- Negatively impacts the Media sector but has no significant effect on other sectors.
- This could be due to increased energy costs reducing corporate profitability.

- Exchange Rate (ER):

- Does not significantly impact any sectoral index.
- Suggests that stock prices in India are not strongly influenced by exchange rate fluctuations.

 

1. **Short-Run Relationship (Error Correction Model Results)**

- Foreign Institutional Investment (FII):

- A key determinant of short-run market movements across multiple sectors.

- Exchange Rate (ER):

- Shows short-term negative effects on multiple sectors but lacks long-term significance.

- Crude Oil Price (COP):

- Impacts the Banking and Financial Services sectors in the short run.

 

1. **Model Diagnostics and Reliability**

- The Ramsey RESET test confirms that the models are correctly specified.
- The Breusch-Pagan-Godfrey test shows no issue of heteroskedasticity.
- The Breusch-Godfrey serial correlation LM test confirms no serial correlation in residuals.
- The CUSUM plot indicates that the model is stable over time.

**Conclusion & Implications:**

- **Key Takeaways:**

- FII, IIP, EPU, MS, and WPI are the most significant long-term determinants of stock market returns.
- In the short run, FII, ER, and COP are the primary factors influencing stock returns.
- The impact of macroeconomic indicators varies significantly across different sectors.

- **Implications for Investors:**

- Investors should consider sectoral differences when making decisions.
- Foreign investment trends and liquidity conditions (money supply) play crucial roles in market movements.
- Inflation and industrial production can positively affect certain sectors like FMCG and Auto.

- **Implications for Policymakers:**

- Policymakers should monitor foreign institutional flows, as they significantly impact stock market volatility.
- Economic policy reforms can have long-term positive effects on specific sectors like Auto and Pharma.
- Exchange rate fluctuations do not significantly affect sectoral indices, indicating that the Indian stock market is somewhat insulated from currency shocks.

**Final Thoughts:**

 This study provides valuable insights into how macroeconomic indicators influence sector-specific stock market performance in India. The findings can guide investment strategies, portfolio diversification, and economic policymaking by highlighting sector-specific sensitivities to economic factors.

**Explanation to the answers:**

**Is the work clearly and accurately presented and does it cite the current literature?**

**Partly**

**Justification for the answer:**

 While the study is well-structured and presents its findings clearly, there are some areas where clarity could be improved, particularly in explaining certain econometric techniques for a broader audience. Additionally, although the paper references relevant literature, some recent studies on sectoral stock market responses to macroeconomic indicators could have been included to enhance the discussion.

**Is the study design appropriate and is the work technically sound?**

**Yes**

**Justification for the answer: **

1. **Appropriateness of Study Design:**

- The study adopts a quantitative econometric approach, which is highly suitable for analysing the relationship between macroeconomic indicators and sectoral stock market performance.
- The Autoregressive Distributed Lag (ARDL) model is an appropriate choice for this study because it allows for the examination of both short-run and long-run relationships, even when variables are integrated at different levels (I(0) and I(1)).
- The use of monthly data spanning over 12 years (April 2012 – August 2024) provides a robust sample size, ensuring that findings are reliable and not overly influenced by short-term fluctuations.

1. **Technical Soundness:**

- The study follows a rigorous econometric methodology with necessary statistical checks, including: Stationarity tests (Augmented Dickey-Fuller test) to determine integration order. Cointegration analysis (ARDL bounds test) to establish long-run relationships. Error Correction Model (ECM) to account for short-run dynamics.
- Diagnostic tests such as the Breusch-Pagan-Godfrey test (for heteroskedasticity), Breusch-Godfrey serial correlation LM test, and Ramsey RESET test confirm that the model is well-specified and free from major econometric issues.
- The CUSUM test for model stability further strengthens confidence in the findings.

1. **Robustness and Reliability:**

- The study carefully differentiates between sectoral responses, acknowledging that macroeconomic factors do not uniformly impact all industries.
- The results are interpreted logically and align with established economic theories, enhancing their credibility.
- The inclusion of multiple macroeconomic indicators (e.g., Crude Oil Price, FII, Exchange Rate, Money Supply, and Economic Policy Uncertainty) ensures a comprehensive evaluation of market influences.

**Are sufficient details of methods and analysis provided to allow replication by others?**

**Yes**

**Justification for the answer:**

- The study clearly outlines the data sources, specifying the macroeconomic indicators and sectoral indices used, along with their respective time periods (April 2012 – August 2024).
- The econometric methodology is well-documented, including stationarity tests (ADF test), ARDL model specification, bounds testing, and the error correction model (ECM).
- The equations for statistical models are explicitly stated, making it easier for other researchers to replicate the analysis.
- Diagnostic tests (Breusch-Pagan-Godfrey, Breusch-Godfrey LM, Ramsey RESET, CUSUM) are reported, ensuring transparency in model validation.

In view of the above points, the study provides sufficient methodological details, including data sources, statistical models, and validation tests, making it fully replicable by other researchers.

**If applicable, is the statistical analysis and its interpretation appropriate?**

 Yes

**Justification for the answer:**

- The study applies appropriate econometric techniques, including the ARDL model, which is well-suited for analyzing both short-run and long-run relationships between macroeconomic indicators and sectoral indices.
- Stationarity checks (ADF test) and cointegration analysis (ARDL bounds test) ensure that the data meet the necessary assumptions for time-series modeling.
- The Error Correction Model (ECM) is effectively used to capture short-run adjustments.
- Diagnostic tests (e.g., Breusch-Godfrey LM, Breusch-Pagan-Godfrey, Ramsey RESET, and CUSUM stability test) confirm the robustness of the statistical analysis.

**Are all the source data underlying the results available to ensure full reproducibility?**

**Yes**

**Justification for the answer: **

- The study clearly specifies the data sources for macroeconomic indicators and sectoral indices, including Investing.com, RBI, MOSPI, and FRED, ensuring transparency.
- The dataset spans April 2012 to August 2024, providing a well-documented timeframe for replication.
- The use of publicly available economic and financial data ensures that other researchers can access the same dataset for verification.

**Are the conclusions drawn adequately supported by the results?**

**Yes**

**Justification for the answer: **

- The conclusions align well with the empirical findings, demonstrating sector-specific effects of macroeconomic indicators.
- The ARDL model results clearly distinguish between short-run and long-run relationships, reinforcing the study’s key insights.
- The statistical tests validate the robustness of the results, ensuring that the interpretations are data-driven.

Is the work clearly and accurately presented and does it cite the current literature?

Partly

If applicable, is the statistical analysis and its interpretation appropriate?

Yes

Are all the source data underlying the results available to ensure full reproducibility?

Yes

Is the study design appropriate and is the work technically sound?

Yes

Are the conclusions drawn adequately supported by the results?

Yes

Are sufficient details of methods and analysis provided to allow replication by others?

Yes

Reviewer Expertise:

Finance and Accounts; Marketing Management; Entrepreneurship

I confirm that I have read this submission and believe that I have an appropriate level of expertise to confirm that it is of an acceptable scientific standard.

# Response

**Published:** 2025-03-30

**Authors:** Sanjay Chauhan

Respected Sir

 some recent studies on relationship between macroeconomic variables and sectoral indices are added in the new version.

 Thank You

# Reviewer report: Reviewer response for version 1

**DOI:** 10.5256/f1000research.176599.r367781

**Published:** 2025-02-27

**Authors:** Werner R Murhadi

**Affiliations:** 1 Universitas Surabaya, Surabaya, East Java, Indonesia

This research is exciting and has the potential to contribute significantly to the understanding of macroeconomic factors that impact each sector. The researcher explained the research objectives, literature review, and methods used well. This study examines the impact of macroeconomic factors such as foreign institutional investment, the index of industrial production, money supply, the wholesale price index, the economic policy uncertainty index, crude oil prices, and exchange rates. The research examined the macroeconomic influence on the performance of 9 sectors: automotive, bank, financial service, FMCG, IT, Media, Metal, Pharmacy, and Realty.

**Suggestion: Based on Table 5. Long-run form of ARDL.**

 - Based on the research results, Foreign Institutional Investment (FII) significantly affects all the sectoral indices except IT. This result is interesting because FII does not affect the IT sector index. IT investment requires significant funding and is mainly expected from foreign direct investment. Why FII does not affect the IT sector needs to be elaborated on.

 - The index of industrial production (IIP) has a significant relationship with Auto, IT, Media, Metal and Pharma. As we know, The Index of Industrial Production (IIP) is an index that indicates the performance of various industrial sectors of the Indian economy. It is interesting to explain why this index does not affect the FMCG sector, which is one of the sectors that contributes heavily to industrial production.

 - Money supply (MS) significantly affects Banks, FMCG, and IT in the long run. The results of this study are interesting in that MS negatively affects the performance of the Bank and FMCG sectors but positively affects the IT sector. An explanation of why MS can positively affect the IT sector will add insight for readers and observers.

 - Crude oil price (COP) significantly affects Media in the long run. This result is fascinating, as COP has a negative effect on the media sector and no effect on other sectors. An explanation needs to be added as to why COP negatively affects the media sector and does not affect the auto, FMCG, metal, pharma, and real estate sectors, which are naturally large energy users.

 - Exchange rate (ER) does not significantly affect the sectoral index. This result needs further explanation for why ER does not affect all sectors. Generally, some sectors use large imported goods such as auto, FMCG, Metal, and Pharmacy. It needs to be explained why the exchange rate fluctuations in the long run do not affect the performance of these sectors.

Is the work clearly and accurately presented and does it cite the current literature?

Yes

If applicable, is the statistical analysis and its interpretation appropriate?

Yes

Are all the source data underlying the results available to ensure full reproducibility?

Yes

Is the study design appropriate and is the work technically sound?

Yes

Are the conclusions drawn adequately supported by the results?

Yes

Are sufficient details of methods and analysis provided to allow replication by others?

Yes

Reviewer Expertise:

Financial and strategic management

I confirm that I have read this submission and believe that I have an appropriate level of expertise to confirm that it is of an acceptable scientific standard, however I have significant reservations, as outlined above.

# Response

**Published:** 2025-03-30

**Authors:** Sanjay Chauhan

Respected Sir

 All the suggestions are addressed in the new version.

 Thank You

# Response

**Published:** 2025-04-10

**Authors:** Werner Murhadi

**Affiliations:** Management, University of Surabaya, Surabaya, East Java, Indonesia

After I checked for suggestions to make it easier for readers to understand this article, we have not found the requested explanation below:

 - Based on the research results, Foreign Institutional Investment (FII) significantly affects all the sectoral indices except IT. This result is interesting because FII does not affect the IT sector index. IT investment requires significant funding and is mainly expected from foreign direct investment. Why FII does not affect the IT sector needs to be elaborated on.

 - The index of industrial production (IIP) has a significant relationship with Auto, IT, Media, Metal and Pharma. As we know, The Index of Industrial Production (IIP) is an index that indicates the performance of various industrial sectors of the Indian economy. It is interesting to explain why this index does not affect the FMCG sector, which is one of the sectors that contributes heavily to industrial production.

 - Money supply (MS) significantly affects Banks, FMCG, and IT in the long run. The results of this study are interesting in that MS negatively affects the performance of the Bank and FMCG sectors but positively affects the IT sector. An explanation of why MS can positively affect the IT sector will add insight for readers and observers.

 - Crude oil price (COP) significantly affects Media in the long run. This result is fascinating, as COP has a negative effect on the media sector and no effect on other sectors. An explanation needs to be added as to why COP negatively affects the media sector and does not affect the auto, FMCG, metal, pharma, and real estate sectors, which are naturally large energy users.

 - Exchange rate (ER) does not significantly affect the sectoral index. This result needs further explanation for why ER does not affect all sectors. Generally, some sectors use large imported goods such as auto, FMCG, metal, and pharmacy. It needs to be explained why the exchange rate fluctuations in the long run do not affect the performance of these sectors.

 We hope that the article will not only explain that the research results support the findings of researcher x or do not support the findings of researcher Y, but readers will find it easier to understand if there is an explanation of why there are findings such as those referred to above?

 Translated with DeepL.com (free version)
